# Peptide Vaccines for Hypertension and Diabetes Mellitus

**DOI:** 10.3390/vaccines2040832

**Published:** 2014-11-26

**Authors:** Hironori Nakagami, Hiroshi Koriyama, Ryuichi Morishita

**Affiliations:** 1 Division of Vascular Medicine and Epigenetics, Osaka University United Graduate School of Child Development, Osaka University, Kanazawa University and Hamamatsu University School of Medicine, Chiba University, Fukui University, 2-1 Yamada-oka, Suita, Osaka 565-0871, Japan; E-Mail: koriyama@cgt.med.osaka-u.ac.jp; 2 Department of Clinical Gene Therapy, Graduate School of Medicine, Osaka University, 2-2 Yamada-oka, Suita, Osaka 565-0871, Japan; E-Mail: morishit@cgt.med.osaka-u.ac.jp

**Keywords:** vaccine, angiotensin II, DPP4

## Abstract

Vaccines are commonly used as a preventive medicine for infectious diseases worldwide; however, the trial for an amyloid beta vaccine against Alzheimer’s disease will open a new concept in vaccination. In case of therapeutic vaccines for cancer, their targets are usually specific antigens in cancer cells, allowing activated cytotoxic T cells (CTLs) to attach and remove the antigen-presenting cancer cells. In our therapeutic vaccines against hypertension, the target is angiotensin II (Ang II) and induced anti-Ang II antibodies could efficiently ameliorate high blood pressure. Similarly, we developed the therapeutic vaccine against DPP4 for diabetes mellitus. However, because Ang II or DPP4 is an endogenous hormone, we must avoid autoimmune disease induced by these vaccines. Therefore, our system was used to design a therapeutic vaccine that elicits anti-Ang II or DPP4 antibodies without CTL activation against Ang II or DPP4. In this review, we will describe our concept of therapeutic vaccines for hypertension and diabetes mellitus.

## 1. Introduction

Vaccines are commonly used to protect against infectious diseases worldwide; however, the trial for an amyloid beta vaccine against Alzheimer’s disease, cancer and rheumatoid arthritis will open a new concept in vaccination [1,2,3,4,5]. We will pursue the development of a vaccine for patients with high blood pressure or hyperglycemia. The number of patients with hypertension and diabetes mellitus increases each year, and several types of drugs are available for treatment. However, patients often need to take two or three drugs at a time to control severe high blood pressure and hyperglycemia. Therefore, the increased medical cost for hypertension and diabetes mellitus might pose a social problem that affects social security expenses. The ultimate goal of our study is to reduce medical costs through the clinical use of a therapeutic vaccine against hypertension or diabetes mellitus.

## 2. Therapeutic Vaccine for Hypertension

In considering clinical applications, the safety issues of therapeutic vaccines for hypertension and diabetes mellitus should be carefully examined. In the history of therapeutic vaccines, the amyloid beta vaccine effectively reduced amyloid plaques and recovered memory function in several animal models of Alzheimer’s disease [3,4,5]. Unfortunately, however, the clinical trial for this vaccine was halted when 6% of the participants developed aseptic meningoencephalitis, even though amyloid plaque reduction was observed [4,6]. Postmortem examination of the brains of two patients who suffered from aseptic meningoencephalitis due to the vaccine revealed T lymphocyte infiltration into the brain [7,8]. Based on previous experience, therapeutic vaccine must be required to be safe and efficient.

### 2.1. History of Peptide Vaccine for Hypertension

Vaccines for hypertension, targeting the renin-angiotensin system, have been reported since the 1950s [9,10,11,12,13,14,15,16,17,18,19,20,21]. Although a renin vaccine was reported to successfully reduce blood pressure, Michel *et al.* reported that the vaccine induced autoimmune disease of the kidneys in the animal model [19,20]. An angiotensin I vaccine (PMD3117) reduced blood pressure in rat and mouse models, but no human clinical trial [10,11]. Although an angiotensin II (Ang II) vaccine (AngQb-Cyt006) was reported to be effective at producing anti-Ang II antibodies in both rodents [12] and humans [9], further clinical studies of the Ang II vaccine failed to reproduce the blood pressure reduction, despite shorter dosing intervals (0, two, four, six and 10 weeks) and higher antibody titers. Therefore, the development of an Ang II vaccine for clinical application has now been halted. Recently, Liao *et al.* designed the therapeutic vaccine for renin or angiotensin type 1 receptor, and showed the effectiveness of this vaccine in hypertensive rat model [21,22].

### 2.2. Concept of Peptide Vaccine for Hypertension

Our immune system can distinguish self from non-self because the immune system has evolved central and peripheral self-tolerance checkpoints to remove or silence autoreactive lymphocytes. B cells that react to self-antigens become anergic and functionally silent. In order for dormant B cells to proliferate, B cells need to interact with activated T-helper cells recognizing antigen-derived peptides on MHC (major histocompatibility complex) class II molecules presented by B cells. T cells that react with self-antigen, having escaped thymic deletion and existing in peripheral lymphoid organs, may be activated by antigen stimulation in the presence of strong adjuvants. Although our therapeutic vaccine would essentially need to break down peripheral tolerance to induce a reaction against endogenous molecules, the preferred strategy in the development of vaccines against self-antigens is to circumvent T-cell tolerance rather than to break it. The outcome of amyloid beta vaccination provides support for the theory that the adverse effects of the vaccine were due to a T-cell-mediated autoimmune response [4,8,9,10]. This theory also gains support from the presence of a T-cell epitope in the amyloid beta used for immunization, which was considered to be responsible for eliciting autoimmunity. Consequently, the vaccine was modified to exclude T-cell epitopes, therefore avoiding T-cell activation without disrupting the B-cell epitopes responsible for antibody production. While considering this strategy, we speculate that because angiotensin II is only eight amino acids long, which is shorter than amyloid beta (40–42 amino acids long), it might not provide a T-cell epitope. It is known that angiotensin II can provide a B-cell epitope because the existence of anti-angiotensin II antibodies has been confirmed in several experiments. Thus, we will examine whether or not angiotensin II can activate T-cells in our therapeutic vaccine system.

**Figure 1:**
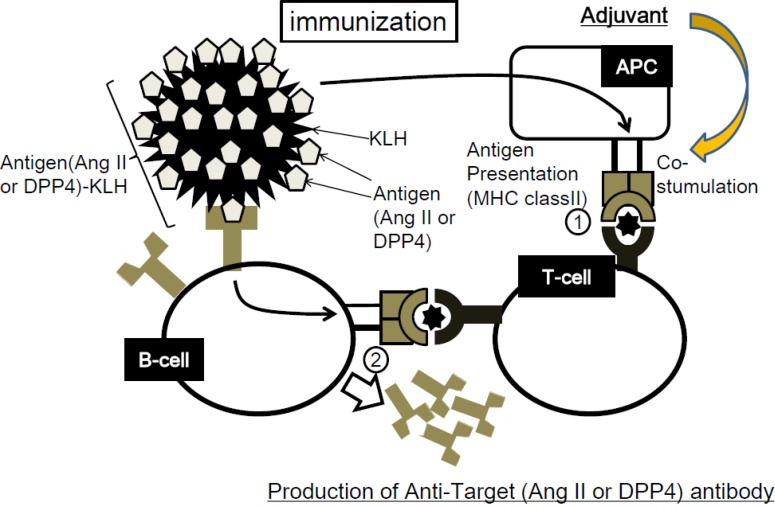
Conceptual schematic of therapeutic vaccine. (Step1) The antigen-presenting cells (APCs) (the antigen, *i.e.*, Ang II or DPP4, in this case is known as a hapten) phagocytose the antigen-KLH conjugate and present a T-cell epitope of KLH to T-cells through the major histocompatibility complex (MHC). T-cells recognize it through T-cell receptors and become activated. (Step2) B-cells specifically recognizing the target antigen differentiate into plasmacytes and proliferate with the help of activated T-cells. Then, B-cells produce anti-angiotensin II (Ang II) or DPP4 antibody.

We show the concept of our therapeutic vaccine in Figure 1. Stable and sufficient antibody production requires helper T-cell activation to assist in the polyclonal expansion of B-cells; therefore, antigens must contain both B-cell and T-cell epitopes. As we want to avoid T-cell epitopes from our target molecule, immunogenic molecules (*i.e.*, KLH) are conjugated with the antigen. Consequently, T-cells would be activated by KLH, instead of the epitopes of our target molecule. We need to confirm that antibodies against our target molecule (*i.e.*, Angiotensin II) are successfully induced after immunization with our target molecule, thereby confirming the existence of a B-cell epitope on our target molecule. As for T-cell epitopes, we will perform T-cell proliferation assays and ELISPOT assays, which may indicate to us the responsiveness of T-cells to our target molecule. Ang II-KLH has been used as an Ang II peptide vaccine in mice. The results indicated that Ang II-KLH and KLH induced T-cell activation but Ang II did not, which means that KLH contains a T-cell epitope, but Ang II does not [23]. Importantly, the sources of T-cell epitopes and B-cell epitopes can be different. This situation is reflected in the relationship between a hapten and its carrier, in which the hapten has the only B-cell epitope and the carrier possesses the T-cell epitope. Based on this mechanism for our therapeutic vaccine system, autoimmune diseases caused by cytotoxic T-cells can be avoided.

### 2.3. Experimental Results of Therapeutic Vaccine for Hypertension

Most anti-hypertensive drugs are safe and effective for patients. Thus, the vaccine against hypertension must be highly safe and effective. We have examined the potential of an Ang II vaccine in mice [23]. The anti-Ang II antibody production of B-cells was evaluated in response to vaccination with Ang II, KLH and Ang II-KLH with or without adjuvant. As a result, only Ang II-KLH with adjuvant successfully induced the production of anti-Ang II IgG antibodies. The antibody titers in the Ang II-KLH with adjuvant group increased as the dose of the antigen increased, and the anti-Ang II antibody recognized angiotensin I (Ang I) to a lesser extent, although it did not recognize angiotensinogen. These results suggest that vaccination with Ang II-KLH and adjuvant can produce IgG antibodies specific for Ang II in C57Bl/6 mice. In addition, we also examined the subtype of IgG (*i.e.*, IgG1, IgG2a, IgG2b, *etc.*) and IgM in this experiment. As a result, we observed the marked elevation of IgG1 and IgM antibodies specific for Ang II (IgG2a is usually not observed in C57Bl/6 mice). We further examined the time course of anti-Ang II antibody titers after vaccination with Ang II-KLH. The antibody titer peaked on day 42 and decreased on days 70 and 98. To examine whether B-cells could be activated by Ang II itself, we compared the antibody titers in mice prior to AngII infusion (day 42) with titers obtained after Ang II infusion (day 56). The post-infusion titer was lower than the pre-infusion titer. In addition, the post-infusion titer decreased in the same manner as in the titer with no Ang II infusion. This result suggests that endogenous Ang II does not stimulate antibody production, even after Ang II has been recognized as a target by immune system. Importantly, after retreatment of Ang II-KLH in immunized mice, we observed the increased anti-Ang II IgG titer, which may be a booster effect. Therefore, we will propose the reinjection of Ang II vaccine two or three times per year to keep the continuous control of blood pressure in the protocol of clinical study.

To evaluate the effect of immunization in mice, we examined the effect of Ang II-KLH vaccination in mice using an Ang II infusion model. Interestingly, at steady-state, mice immunized with Ang II-KLH failed to show lower blood pressure than control mice. After Ang II infusion (1000 ng/kg/min), the systolic blood pressure was increased in the control mice, but not in the high dose-immunized mice. Thus, mice immunized with Ang II-KLH exhibited a significant decrease in systolic blood pressure compared to the control mice. Importantly, a negative correlation was observed between the anti-Ang II antibody titer and systolic blood pressure in the immunized mice after Ang II infusion. This result indicates that the antibody elicited by the vaccine efficiently reduces blood pressure in an Ang II infusion model. Furthermore, we examined the degree of cardiac hypertrophy and fibrosis after Ang II infusion in high-dose immunized mice and control mice. Systemic Ang II treatment caused myocardial hypertrophy in control mice, but immunized mice exhibited an amelioration of the Ang II-induced increase in the heart weight to body weight ratio. Similarly, Ang II treatment led to the development of perivascular fibrosis in the hearts of control mice, but immunized mice exhibited less fibrotic changes [23]. These results revealed that the anti-Ang II antibody induced by Ang II-KLH vaccination efficiently attenuated Ang II-induced signaling *in vitro* and Ang II-induced hypertension and cardiac remodeling *in vivo*.

## 3. Therapeutic Vaccine for Diabetes Mellitus

Glucagon-like peptide-1 (GLP-1), one of the incretin hormones, is critical for glucose homeostasis and represents a therapeutic target for diabetes mellitus [24]. GLP-1 increases insulin secretion and improves insulin sensitivity [25,26] but is rapidly degraded by the enzyme dipeptidyl peptidase-4 (DPP4) [27,28]. DPP4 inhibitors are currently being used clinically in patients with diabetes mellitus [29,30]. We developed a therapeutic vaccine against DPP4 for diabetes mellitus treatment [31].

### 3.1. Design of DPP4 Vaccine

We shortened the antigen of DPP4 as much as possible because the shorten antigen might reduce the risk to include the T-cell epitode in antigen sequence. We selected a few epitopes within DPP4 as antigens which were conjugated to KLH as a carrier protein, and the DPP4 vaccines with adjuvants were administered to mice a total of three times with two-week intervals between injections. Interestingly, one of DPP4 vaccines resulted in an increase of anti-DPP4 antibody titer as well as a decrease of the plasma DPP4 activity.

### 3.2. Experimental Results of Therapeutic Vaccine for Diabetes Mellitus

To evaluate the effect of the DPP4 vaccine on glucose metabolism, we performed a meal tolerance test in mice with high fat diet. In mice with high fat diet beginning one week after the immunization protocol, the glucose level was significantly lower as compared to control groups. The increased plasma insulin levels in mice fed a high fat diet were also decreased following DPP4 vaccination. Moreover, in the homeostasis model assessment for insulin resistance (HOMA-IR), DPP4 vaccine significantly improved insulin sensitivity levels compared to control group. In accordance, in the insulin challenge test, DPP4 vaccine significantly improved the rate of blood glucose reduction compared to control group.

To further investigate the efficacy of the DPP4 vaccine, we utilized a spontaneous diabetic model, the yellow KK mice that carry the obsess gene A^y^ and develop obesity, hyperglycemia and hyperinsulinemia. The immunized mice by DPP4 vaccine exhibited significant improvement in the postprandial blood glucose levels and tended to have increased plasma insulin secretion and GLP-1 level [31].

### 3.3. Evaluation of Safety Issues for DPP4 Vaccine

Peptide vaccines as immunotherapy against self-antigens carry the risk of causing adverse effects. Previous vaccines against amyloid beta led to T-cell-mediated aseptic meningoencephalitis [7]. Therefore, a detailed evaluation of T-cell activation is warranted to support the clinical application of future vaccines. The strategy of using a peptide from a self-antigen conjugated to an appropriate carrier protein has been shown to successfully break tolerance and produce antibodies against the self-antigen. We also evaluated the potential autoimmune response against DPP4, as B- and T-cell epitopes were present in the DPP4 vaccine. Concerning B-cell epitopes, DPP4 vaccine produced a strong titer of antibodies against DPP4 after immunization. We also investigated T-cell activation in DPP4-immunized mice using the T-cell proliferation assay or an enzyme-linked immunosorbent spot (ELISPOT) assay. In the T-cell proliferation assay, stimulation with KLH significantly induced the proliferation of splenocytes isolated from DPP4 vaccinated mice, but stimulation with the DPP4 peptide did not induce proliferation as compared to the unstimulated control. Similarly, in the production of IFN-γ and IL-4 cytokines associated respectively with Th1 and Th2 responses in splenocytes from DPP4 immunized mice by ELISPOT, stimulation with KLH induced the production of IFN-γ and IL-4. However, the DPP4 peptide did not elicit a significant response in comparison to the unstimulated and KLH control groups. These results indicated that KLH contains adequate T-cell epitopes to induce T-cell activation in immunized mice, whereas DPP4 was unable to elicit T-cell activation even after immunization with E3. Thus, this level of T-cell activation was sufficient to promote antibody production but was not capable of inducing an autoimmune response.

We also examined whether the immunized plasma would cause the damages to cells expressing DPP4 through the complement dependent cell cytotoxicity (CDC) mediated processes. For both cytotoxic assays, anti-CD20 antibody is used as a positive control, and splenocytes were selected as target cells, because both CD20 and DPP4 are expressed in cultured cells obtained from spleen. In CDC assay, anti-CD20 antibody caused cell death with co-treatment of complement serum. However, the purified IgG from the mice immunized with DPP4 vaccine did not cause cell death in the same optimized condition. These results suggest that immunized plasma does not cause death in cells expressing DPP4. Furthermore, we also evaluated immune mediated damage in tissues, such as jejunum, liver, and kidney, where endothelial DPP4 is expressed at a high level. Similarly, no clear tissue injury or leucocyte accumulation was observed at the end of the study period in DPP4 immunized mice. These results suggested that the DPP4 vaccine did not have the potential to stimulate an autoimmune response [31].

## 4. Conclusions

We have begun to investigate the development of therapeutic vaccines for lifestyle-related diseases. In the view of hypertension or diabetes therapy, the prolonged effect of therapeutic vaccine is advantage for the tight regulation of blood pressure or blood sugar levels. However, in the case of hypotension or hypoglycemia, this prolonged effect might be a disadvantage because its effect cannot be quickly withdrawn. We have to consider how to rescue the immunotherapy in such a case. Toward clinical application, the design of clinical trial will be important to prove our proof of concept in the clinical setting. To achieve the reduction of medical costs by the clinical use of a therapeutic vaccine, we will address common diseases as well as severe diseases as a target of therapeutic vaccines in future. We hope that this novel concept of this study will contribute to promoting health and medicine in the future.
